# Epidemiology and outcomes of hypoglycemia in patients with advanced diabetic kidney disease on dialysis: A national cohort study

**DOI:** 10.1371/journal.pone.0174601

**Published:** 2017-03-29

**Authors:** Yeh-Wen Chu, Hsuan-Ming Lin, Jhi-Joung Wang, Shih-Feng Weng, Chih-Ching Lin, Chih-Chiang Chien

**Affiliations:** 1 Department of Nephrology, Chi-Mei Medical Center, Tainan, Taiwan; 2 Department of Nephrology, An Nan Hospital, China Medical University, Tainan, Taiwan; 3 Department of Medical Research, Chi-Mei Medical Center, Tainan, Taiwan; 4 Department of Healthcare Administration and Medical Informatics, Kaohsiung Medical University, Kaohsiung, Taiwan; 5 Department of Nephrology, Taipei Veterans General Hospital, Taipei, Taiwan; Hospital Universitario de la Princesa, SPAIN

## Abstract

**Background:**

Patients with advanced diabetic kidney disease (DKD) behave differently to diabetic patients without kidney disease. We aimed to investigate the associations of hypoglycemia and outcomes after initiation of dialysis in patients with advanced DKD on dialysis.

**Methods:**

Using National Health Insurance Research Database, 20,845 advanced DKD patients beginning long-term dialysis between 2002 and 2006 were enrolled. We investigated the incidence of severe hypoglycemia episodes before initiation of dialysis. Patients were followed from date of first dialysis to death, end of dialysis, or 2008. Main outcomes measured were all-cause mortality, myocardial infarction (MI), and subsequent severe hypoglycemic episodes after dialysis.

**Results:**

19.18% patients had at least one hypoglycemia episode during 1-year period before initiation of dialysis. Advanced DKD patients with higher adapted Diabetes Complications Severity Index (aDCSI) scores were associated with more frequent hypoglycemia (P for trend < 0.001). Mortality and subsequent severe hypoglycemia after dialysis both increased with number of hypoglycemic episodes. Compared to those who had no hypoglycemic episodes, those who had one had a 15% higher risk of death and a 2.3-fold higher risk of subsequent severe hypoglycemia. Those with two or more episodes had a 19% higher risk of death and a 3.9-fold higher risk of subsequent severe hypoglycemia. However, previous severe hypoglycemia was not correlated with risk of MI after dialysis.

**Conclusions:**

The rate of severe hypoglycemia was high in advanced DKD patients. Patients with higher aDCSI scores tended to have more hypoglycemic episodes. Hypoglycemic episodes were associated with subsequent hypoglycemia and mortality after initiation of dialysis. We studied the associations and further study is needed to establish cause. In addition, more attention is needed for hypoglycemia prevention in advanced DKD patients, especially for those at risk patients.

## Introduction

The increasing prevalence of diabetes mellitus (DM) is a worldwide public health issue [1]. Long-term complications of DM develop gradually with time. Patients with diabetic kidney disease (DKD) make up 40–50% of patients on chronic dialysis [2]. Advanced DKD is not only a risk for hypoglycemia, but also increases the severity of hypoglycemia [3].

The Diabetes Control and Complications Trial, which tested the “Glucose Hypothesis”, found that microvascular and macrovascular disease can be reduced in patients with type 1 DM with early intensive therapy with treatment aiming for a glycated hemoglobin of 7% [4]. The United Kingdom Prospective Diabetes Study also demonstrated that using intensive therapy had long-term benefits in patients with incident type 2 DM (T2DM) [5]. However, the Action to Control Cardiovascular Risk in Diabetes Study (ACCORD) found that lowing glycated hemoglobin to 6.4% increased mortality and had no effect in reducing major cardiovascular events in T2DM patients with diabetic complication compared to those receiving standard therapy. Further analysis found intensive glycaemia control increased hypoglycaemia [6]. Retrospective epidemiological analysis of data from the ACCORD trial revealed severe hypoglycaemia increased risk of death, regardless of the intensity of glucose control [7].

Patients with advanced DKD on dialysis behave differently to DM patients without kidney disease. More than forty percent of patients with advanced DKD have severe hypoglycemia [8]. Both DKD and hypoglycemia are associated with increased morbidity and mortality [9]. Re-analysis of the ACCORD trial revealed higher risk of hypoglycemia in DKD, regardless of intensive and standard group [10]. Patients with advanced DKD usually have multiple comorbid diabetic complications. In addition, DKD is associated with an increased risk of cardiovascular (CV) events. The increase in CV risk is multifactorial: not only traditional CV risks, but also novel and uremic CV risks with kidney function deterioration. To the best of our knowledge, no study has been undertaken to evaluate the association of hypoglycemia and long-term outcomes in patients with advanced DKD. Therefore, using a large data set from the Taiwan`s National Health Insurance Research Database (NHIRD), we evaluated the association of severe hypoglycemia and outcomes after initiation of dialysis in patients with advanced DKD. Outcomes included all-cause mortality, subsequent hypoglycemia, and myocardial infarction (MI) after dialysis.

## Materials and methods

### Database

Since Taiwan`s National Health Insurance (NHI) program was implemented in 1995, it has provided compulsory universal health insurance to all of Taiwan`s residents with the exception of prison inmates. All medical institutions in contract with the NHI program must submit standard computerized claim documents for reimbursement of medical expenses. Patients with end-stage renal disease (ESRD) are eligible for any type of renal replacement therapy free of charge. All chronic dialysis patients are covered by NHI.

Data were obtained from Taiwan`s National Health Insurance Research Database (NHIRD) [Bureau of National Health Insurance. This database is managed by Taiwan`s National Health Research Institute who released the data to researchers. The NHIRD, which contains data from nearly all (99%) inpatient and outpatient medical benefit claims made for Taiwan’s 23 million residents, is one of the largest and most comprehensive databases in the world, and has been used extensively in various studies [available at http://nhird.nhri.org.tw/ (In Chinese) and http://nhird.nhri.org.tw/en/How_to_cite_us.html (In English)]. Gender, birthday, dates of admission and discharge, medical institutions providing the services, diagnostic and procedure codes (up to five each), and outcomes are encrypted. This study tapped the NHIRD to gain access to ambulatory care claims data, inpatient claims, the Catastrophic Illness Database (CID) and updated beneficiary registry data. As the dataset was released with deidentified secondary data for public research purposes, the study was exempt from full review by the Institutional Review Board of Chi-Mei medical center and the Bureau of National Health Insurance (NHRINHIRD-99182).

In Taiwan, the NHI Bureau issues major illness/injury certificates to all patients who had ESRD needed received long-term dialysis. So, ESRD needed received long-term dialysis is among the listed catastrophic illness in the CID in Taiwan. Individuals who are registered in the CID for ESRD needed received long-term dialysis must provide the NHI Administration review board a physician`s diagnosis certificate and relevant medical records, including dialysis information. ESRD need received long-term dialysis was validated through an expert review process. When the applications are approved, the patients are exempted from disease-related copayments for medical services, including the dialysis fees.

### Patient selection and definition

For this longitudinal cohort study, we first identified adult ESRD patients (≥18 years old) on maintenance dialysis beginning between January 1, 2002 and December 31, 2006. Maintenance dialysis was defined as having received dialysis for more than 90 days. A total of 39,956 incident ESRD dialysis patients were identified. Sixty-seven patients without birth date information were excluded. The International Classification of Diseases, 9th Revision, Clinical Modification (ICD-9-CM) codes used to define DM: 250.xx, 357.2, 362.0x, 366.41. Patients with DM were identified according to one of the definitions below: (1) diagnostic codes from outpatient visits if the patient had an initial diagnosis at any time in the 1 year before the start of dialysis and then had one or more additional diagnoses within the subsequent 12 months. The first and last outpatient visit within 1 year had to have been >30 days apart to avoid accidental inclusion of miscoded patients; (2) diagnostic codes in hospitalization databases at least once in the 1 year before the start of dialysis. Among the remaining 39, 889 incident ESRD dialysis patients, 20,883 had DM. Next, patients who had undergone renal transplantation before beginning dialysis (n = 38) were excluded. Finally, this study analyzed data collected from 20, 845 adult advanced DKD patients needed long-term dialysis. Severe hypoglycemia was defined as requiring medical assistance; these patients were identified by a listing of one of the following ICD-9-CM codes: 250.8 (diabetic hypoglycemia, hypoglycemic shock), 251.0 (hypoglycemic coma), 251.1 (hyperinsulinism hypoglycemia) or 251.2 (unspecified hypoglycemia) in the emergency department or inpatient databases any time during the one-year period leading up to beginning of dialysis. We also collected patient demographic data, survival status, date of death, adapted Diabetes Complications Severity Index (aDCSI) score [11–13] and baseline comorbidities.

### Outcomes

The following outcomes were considered: all-cause mortality, subsequent severe hypoglycemia on dialysis within the one-year period following initiation of dialysis, and MI. Antecedent hypoglycemia can blunt subsequent counterregulatory response to hypoglycemia in a short-term period [14]. Moreover, previous hypoglycemia is a risk factor of subsequent hypoglycemia. Yun et al. reported 22.4% of patients with severe hypoglycemia experienced previous hypoglycemic events within 3 months [15]. Some evidences showed hypoglycemia in the previous 6 months was associated with recurrent hypoglycemia in T2DM patients [16, 17]. HYPOS-1 study demonstrated 16.5% T1DM patients experienced severe hypoglycemia in the past 12 months and 78.6% patients had at least one hypoglycemia in the past 4 weeks [18]. To our best of knowledge, no longer interval was studied in the literature. Therefore, we choose 1-year period to investigate the relationship of previous hypoglycemia and subsequent episode. Patients were followed-up from the first reported date of dialysis to the date of death, end of dialysis, or December 31, 2008, whichever occurred first.

### Statistical analyses

Baseline characteristics between the groups who had hypoglycaemic episodes and those who had not were compared using a parametric Pearson’s chi-square test. Significance was set at p < 0.05. The cumulative proportion of survivors after the initiation of dialysis were calculated using the Kaplan-Meier method. The log rank test was used to analyze significance. Cox proportional hazards models were used to identify the risk factors of mortality after the initiation of dialysis. Hazard ratios (HRs) and 95% confidence intervals (CIs) were derived from Cox proportional hazards models. Cox models met the assumption of proportionality of risks. The independent associations were examined using multivariate analysis. All statistical operations were performed using the Statistical Package for Social Sciences for Windows 17.0 (SPSS Inc; Chicago, IL, USA).

## Results

### Patient characteristics

We enrolled 20,845 adult advanced DKD patients started receiving dialysis between 2002 and 2006. Among these patients, 19849 (95.2%) received hemodialysis (HD) and 996 (4.8%) received peritoneal dialysis (PD). Of these patients, 16,847 had no episode of severe hypoglycemia at any time within 1-year period before starting dialysis, 3,019 (14.48%) patients had one episode of severe hypoglycemia, and the other 979 (4.70%) patients had 2 or more episodes (Table 1). Female patients had a higher incidence of hypoglycemia than male patients. Baseline comorbidities included congestive heart failure (CHF), coronary artery disease (CAD), cerebrovascular accident (CVA), dysrhythmia, other cardiac diseases, peripheral vascular disease (PVD), chronic obstructive lung disease (COPD), gastrointestinal (GI) bleeding, liver disease and cancer, all factors relevant to survival in ESRD patients on dialysis [19, 20]. Because all of our patients were advanced DKD on maintenance dialysis, the minimum of aDCSI score was 2. Advanced DKD patients with higher aDCSI scores tended to have a higher frequency of hypoglycemia (P for trend < 0.001). 9.4%, 11.6%, 12.2%, and 17.3% of patients with aDCSI scores 2, 3, 4, and ≥ 5, respectively, had one episode of severe hypoglycemia. In addition, 2.1%, 3.1%, 3.3%, and 6.3% of the patients with aDCSI scores 2, 3, 4, and ≥ 5 had two or more episodes (p< 0.001). Patients on HD had a higher incidence of severe hypoglycemia with 1-year before starting dialysis than those on PD (P<0.001). 3850 (19.4%) of patients on HD and 148 (14.9%) of those on PD had severe hypoglycemia episodes. Patients with severe hypoglycemia tended to have more comorbidities, particularly CHF, CVA, COPD and GI bleeding.

**Table 1:** The baseline characteristics of diabetic end-stage renal disease dialysis patients by hypoglycemic episodes status.

	Without Hypoglycemia (n = 16847)		With 1 hypoglycemic episode (n = 3019)		With ≥2 hypoglycemic episodes (n = 979)		P
	n	(%)	n	(%)	n	(%)	
Sex							<0.001
Female	8257	(79.5)	1573	(15.1)	553	(5.3)	
Male	8590	(82.1)	1446	(13.8)	426	(4.1)	
Age (years)							0.05
18–44	1068	(80.8)	172	(13)	82	(6.2)	
45–64	8079	(80.8)	1451	(14.5)	470	(4.7)	
≥ 65	7700	(80.9)	1396	(14.7)	427	(4.5)	
aDCSI score							<0.001
2	2440	(88.4)	260	(9.4)	59	(2.1)	
3	1935	(85.3)	264	(11.6)	70	(3.1)	
4	3952	(84.5)	573	(12.2)	153	(3.3)	
≥ 5	8520	(76.5)	1922	(17.3)	697	(6.3)	
Congestive heart failure							<0.001
No	10007	(82.2)	1677	(13.8)	493	(4)	
Yes	6840	(78.9)	1342	(15.5)	486	(5.6)	
Coronary artery disease							0.114
No	9799	(81.2)	1738	(14.4)	537	(4.4)	
Yes	7048	(80.4)	1281	(14.6)	442	(5)	
Cerebrovascular disease							<0.001
No	12559	(82)	2094	(13.7)	664	(4.3)	
Yes	4288	(77.6)	925	(16.7)	315	(5.7)	
Dysrhythmia							0.109
No	15006	(80.6)	2712	(14.6)	890	(4.8)	
Yes	1841	(82.3)	307	(13.7)	89	(4)	
Other cardiac^a^							0.349
No	14295	(80.9)	2562	(14.5)	814	(4.6)	
Yes	2552	(80.4)	457	(14.4)	165	(5.2)	
Peripheral vascular disease							0.349
No	15314	(81.1)	2699	(14.3)	859	(4.6)	
Yes	1533	(77.7)	320	(16.2)	120	(6.1)	
Chronic obstructive lung disease							<0.001
No	13876	(81.4)	2426	(14.2)	747	(4.4)	
Yes	2971	(78.3)	593	(15.6)	232	(6.1)	
Gasrointestinal bleeding							<0.001
No	10749	(81.6)	1858	(14.1)	567	(4.3)	
Yes	6098	(79.5)	1161	(15.1)	412	(5.4)	
Liver disease							0.05
No	14321	(81)	2563	(14.5)	804	(4.5)	
Yes	2526	(80)	156	(14.4)	175	(5.5)	
Cancer							<0.001
No	15636	(80.5)	2842	(14.6)	934	(4.8)	
Yes	1211	(84.5)	177	(12.4)	45	(3.1)	

aDCSI = adapted Diabetes Complications Severity Index;

^a^ Includes pericarditis, endocarditis, myocarditis, other complications of heart disease, heart transplant, heart valve replacement, and cardiac devices.

### Risk factors for all-cause mortality following the initiation of dialysis

**Fig 1:**
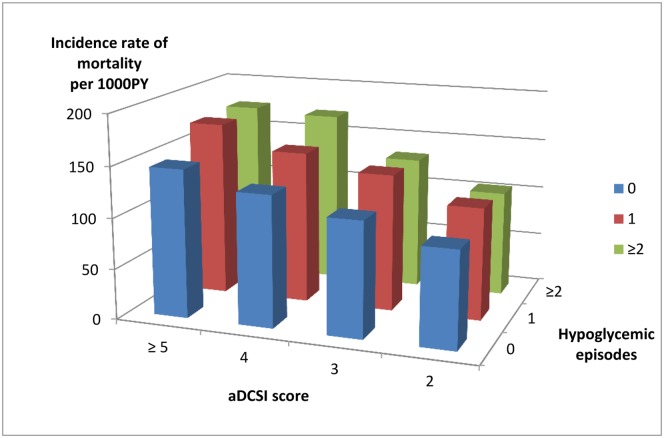
Incidence of mortality after the initiation of dialysis stratified by adapted Diabetes Complications Severity Index (aDCSI) score and hypoglycemic episodes.

During a mean follow-up period of 3.08 ± 1.67 years after dialysis, 8,732 (41.9%) had died. We stratified these DKD patients by aDCSI score and hypoglycemic episodes to investigate their influence on clinical outcomes. Mortality tended to rise along with increase in number of previous hypoglycemic episodes and increases in aDCSI scores (Fig 1). Among patients that had not previously severe hypoglycemia episode, 791 (32.4%), 722 (37.3%), 1585 (40.1%) and 3760 (44.1%) of those with aDCSI scores 2, 3, 4, and ≥ 5 died of any cause. Among those that had one episode of severe hypoglycemia, 91 (35%), 115 (43.6%), 256 (44.7%) and 944 (49.1%) of those with aDCSI scores 2, 3, 4, and ≥ 5 died of any cause. Among those that had two or more episodes of severe hypoglycemia, 20 (33.9%), 30 (42.9%), 73 (47.7%) and 345 (49.5%) of those with aDCSI scores 2, 3, 4, and ≥ 5 died of any cause.

After adjustment, patients had severe hypoglycemia episodes were at a higher risk of mortality (Table 2). Compared to those with no previous severe hypoglycemic episodes, those with one episode had a 15% higher death risk and those with two or more had a 19% higher death risk. Mortality risk also increased 10% with each 1-point increment in aDCSI score.

**Table 2:** Risk factor for all-cause mortality, subsequent severe hypoglycemia on dialysis (within 1-year period after the initiation of dialysis), and Myocardial Infarction (MI).

Covariate	HR (95% CI) for all-cause mortality	HR (95% CI) for Subsequent severe hypoglycemia	HR (95% CI) for myocardial infarction
Sex (Male v Female)	1.080 (1.035–1.125)*	0.989 (0.908–1.078)	1.108 (0.994–1.234)
Age (year; each increment of 1 year)	1.041 (1.039–1.043)*	1.001 (0.997–1.005)	1.024 (1.019–1.030)*
aDCSI score (each increment of 1 score)	1.104 (1.092–1.116)*	1.117 (1.094–1.140)*	1.135 (1.105–1.166)*
Hypoglycemic episodes			
No	1	1	1
1	1.146 (1.082–1.215)*	2.279 (2.060–2.522)*	1.065 (0.918–1.237)
≥ 2	1.194 (1.186–1.312)*	3.903 (3.429–4.441)*	0.904 (0.691–1.182)

* HR adjusted for sex, age, hypoglycemic episodes, aDCSI score and extra—comorbidities (except comorbidities in aDCSI score, including chronic obstructive lung disease, gasrointestinal bleeding, liver disease and cancer).

aDCSI = adapted Diabetes Complications Severity Index

### Risk factors for severe hypoglycemia within 1-year following the initiation of dialysis

**Fig 2:**
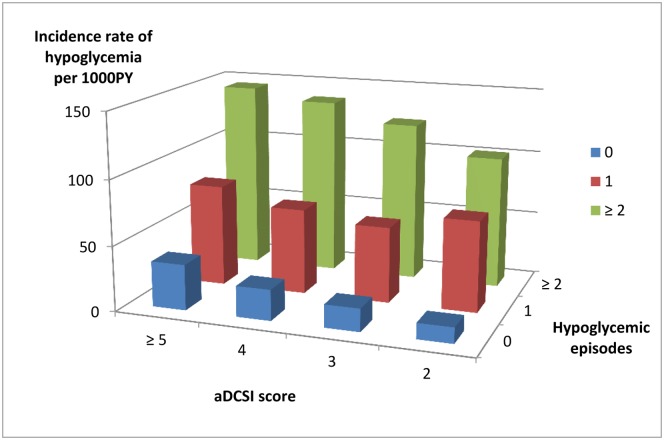
Incidence of subsequent severe hypoglycemia on dialysis (within 1-year period after the initiation of dialysis) stratified by adapted Diabetes Complications Severity Index (aDCSI) score and hypoglycemic episodes.

We studied the incidence of subsequent severe hypoglycemic episodes within 1-year following the initiation of dialysis. The incidence rate of subsequent severe hypoglycemia on dialysis increased along both with increases in previous hypoglycemic episodes and increases in sDCSI scores (Fig 2). In patients that had not previously severe hypoglycemic episode, 100 (4.1%), 107 (5.5%), 274 (6.9%) and 837 (9.8%) of those with aDCSI scores 2, 3, 4, and ≥ 5 had a subsequent severe hypoglycemic episode within 1-year following the initiation of dialysis. Among those who had one severe hypoglycemic episode, 49 (18.8%), 43 (16.3%), 97 (16.9%) and 355 (18.5%) of those with aDCSI scores 2, 3, 4, and ≥ 5 had a subsequent severe hypoglycemic episode. Of those who had two or more severe hypoglycemic episodes, 15 (25.4%), 21 (30%), 45 (29.4%) and 211 (30.3%) of those with aDCSI scores 2, 3, 4, and ≥ 5 had subsequent severe hypoglycemia on dialysis. We further analyzed different dialysis modalities and hypoglycemia. Patients on HD had a higher incidence of severe hypoglycemia within 1-year following the initiation of dialysis; 2078 (10.5%) of patients on HD and only 76 (7.6%) of those on PD had severe hypoglycemia episodes (P<0.004).

After multivariate adjustment, patients with previous severe hypoglycemia episodes were found to be at a higher risk of subsequent severe hypoglycemia on dialysis (Table 2). Compared to those with no previously severe hypoglycemic episodes, those with one episode had a 2.28-fold higher subsequent severe hypoglycemia risk and those with 2 or more a 3.90-fold higher subsequent severe hypoglycemia risk. Subsequent severe hypoglycemia risk also increased 12% with each increment of 1 aDCSI score.

### Risk factors for subsequent myocardial infarction following the initiation of dialysis

**Fig 3:**
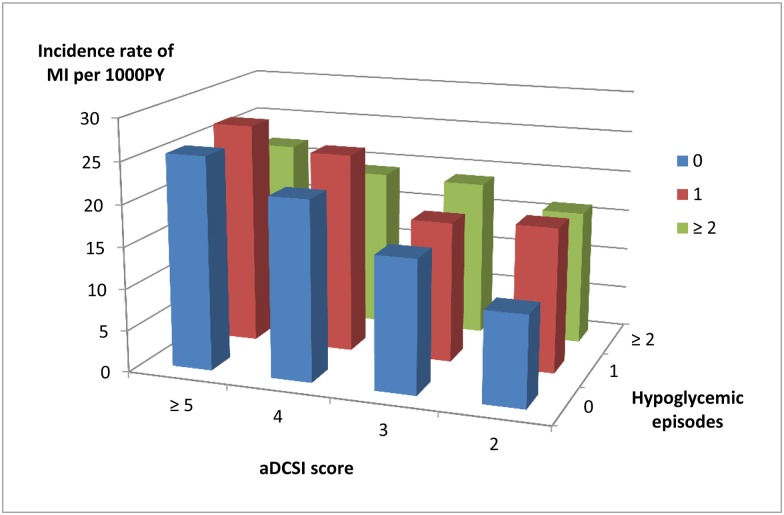
Incidence of myocardial infarction after the initiation of dialysis stratified by adapted Diabetes Complications Severity Index (aDCSI) score and hypoglycemic episodes.

We analyzed the risk for subsequent MI in DKD patients who had begun dialysis. Among those who had no previously severe hypoglycemic episode, 89 (3.6%), 99 (5.1%), 258 (6.4%) and 636 (7.5%) of patients with aDCSI scores 2, 3, 4, and ≥ 5 patients had a MI on dialysis. Among those who had one episode of severe hypoglycemia, 14 (5.4%), 14 (5.3%), 40 (7%) and 140 (7.4%) of patients with aDCSI scores 2, 3, 4, and ≥ 5 had a MI on dialysis. Among those with two or more episodes of severe hypoglycemia, 3 (5.1%), 4 (5.7%), 8 (5.2%) and 42 (6.0%) of patients with aDCSI scores 2, 3, 4, and ≥ 5 had a MI on dialysis (Fig 3).

After multivariate adjustment, patients who had severe hypoglycemia episodes previously were not found to be at greater risk of MI on dialysis (Table 2). However, the risk of MI increased 14% with each one point increment in aDCSI score (HR: 1.14, 95% CI: 1.11–1.17).

## Discussion

This is the first national cohort study to evaluate the associations of severe hypoglycemia and outcomes after dialysis in advanced DKD patients. DM Patients with advanced kidney disease behave differently to those without kidney disease. We found 19.18% of advanced DKD patients had at least one episode of hypoglycemia the year leading up to the beginning of their dialysis therapy. Advanced DKD patients with higher aDCSI scores tended to have more frequent episodes of hypoglycemia. The number of severe hypoglycemic episodes was associated with risk of mortality and subsequent hypoglycemia after dialysis. While, subsequent MI after dialysis was not associated with previous hypoglycemic episodes.

Patients with advanced DKD have a higher risk of hypoglycemia. In another study, only 2% of 15,404 DM patients had at least one episode of hypoglycemia in a 3-year period [21]. The ADVANCE study reported that 2.7% patients in an intensive-control group and 1.5% patients in a standard-control group had at least one severe hypoglycemic episode during a median follow-up of 5 years [22]. The ACCORD study reported that 16.2% and 5.1% of the patients in an intensive therapy group and a standard therapy group, respectively, had hypoglycemia requiring assistance during a mean follow up of 3.5 years [6]. The current study also found DKD patients with higher aDCSI scores tended to have more frequent episodes of hypoglycemia. Hypoglycemia may indicate an underlining health problem [23]. The incidence of hypoglycemia is higher in patients with DKD than those without DKD [3], probably due to the reduction in insulin requirement associated with renal insufficiency [24]. Furthermore, advanced DKD patients have been found to be at greater risk of hypoglycemia in cases involving decreased degradation of insulin in peripheral tissue [25], anorexics with suboptimal nutrition [26], reduced renal gluconeogenesis, and prolonged half-life of antidiabetic drugs. Rates of hypoglycemia have been found to be higher among those with older age, CHF, CAD, CVA, renal failure, autonomic neuropathy, and adrenocortical insufficiency [27–29]. In the current study, older patients and female patients with more comorbidities had higher rates of hypoglycemia.

This study found hypoglycemia to be associated with higher mortality risk in patients with advanced DKD. Hypoglycemia has been reported to be related to increases in one-day mortality in patients with DKD [3]. The ADVANCE study reported severe hypoglycemia to be associated with an increase in death risk [22]. Furthermore, we found an association between frequency of hypoglycemic episodes and increased long-term mortality in ESRD dialysis patients. It is controversial whether the hypoglycemia causes death or whether it is a predictor of underlying problems and poor outcome. After adjustment of aDCSI scores, we found risk of mortality on dialysis increased along with both number of hypoglycemic episodes and increased in aDCSI score. Thus, based on our results, hypoglycemia in DKD patients may not only be a marker of poor outcome, it may also be associated with death. However, because we studies associations, further study is needed to establish cause.

This study found hypoglycemic episodes in DKD to be associated with subsequent hypoglycemia within 1-year following the initiation of dialysis. Previous hypoglycemia is a risk factor associated with further hypoglycemia [30]. We also found increases in aDCSI score to be associated with subsequent hypoglycemia on dialysis. CAD, infection, and poor renal function have been reported to be associated with risks of recurrent hypoglycemia [31]. The HYPOS-1 study reported the rate of hypoglycemia was higher in patients with previous severe hypoglycemia, neuropathy, long duration, and on polypharmacy [18]. Therefore, patients with more comorbidies are at risk for recurrent hypoglycemia and should be closely monitored.

In Taiwan, 95.2% of DM patients with ESRD received HD and only 4.8% received PD. Glucose is one of the contents of hemodialysates [32] and peritoneal dialysates [33]. Different dialysis modalities have different glucose-added dialysates, which maybe an important factor to influence blood glucose. Glucose-added hemodialysates (commonly 100 to 200 mg per deciliter) significantly reduced hypoglycemia in ESRD receiving HD [34, 35]. In addition, higher hemodialysate glucose concentration significantly reduced more hypoglycemia compared with lower hemodialysate glucose concentration [36, 37]. The glucose concentrations of peritoneal dialysates are much higher than those of hemodialysates. In addition, ESRD patients on PD, who received 24-hour continue high-glucose-concentration peritoneal dialysates, can develop hyperglycemia and transient hyperinsulinism [38]. Chinese patients in Hong Kong have a high prevalence of hyperglycemia with daily exchange of 1.5% glucose dialysate [39]. Frequency of hypoglycemia was higher in patients on HD than those on PD [40]. In the current study, we had similar finding. We found 10.5% of DM patients on HD and only 7.6% of those on PD had severe hypoglycemia episodes within 1-year following the initiation of dialysis.

It has been demonstrated hypoglycemic episodes increase CV risk in general population [41–44]. In the current study, we found hypoglycemic episodes in DKD did not correlate with subsequent MI on dialysis. The lack of correlation may be explained by different pathways resulting in CVD in advanced DKD patients. The mechanisms through which DKD may promote CVD include traditional cardiovascular risk factors in general population (e.g., age, male gender, hyperglycemia, DM, hypertension, and smoking) and nontraditional risk factors (e.g., accumulation of uremic toxins, anemia, and disordered mineral metabolism, vascular calcification, hyperhomocysteinemia, inflammation, and oxidative stress) [45, 46].

This study has some limitations. One limitation is that we used ICD-9-CM code to identify various diseases in this study, and thus there is a possibility of misclassification. Additionally, our data were obtained from the NHIRD, a database of insurance claims, which means it lacks laboratory data such as glucose level or glycated hemoglobin. Finally, polypharmacy and anti-diabetic agents are identified as risk factors for hypoglycemia. In HYPOS-1 study, polypharmacy was associated with a 24% excess of risk for hypoglycemia [18]. Hypoglycemia episodes were higher among those with insulin and insulin secretagogues [15, 27]. In addition, angiotensin-converting enzyme inhibitors and beta blockers may contribute to precipitating hypoglycemia by masking the warning symptoms [18]. Therefore, it would be better to describe the data on medications received by subjects; however, there was no information of medications in our database. Further studies needed to be performed to evaluate it.

In conclusion, the rate of severe hypoglycemia was high in advanced DKD patients. Severe hypoglycemic episodes and diabetic complications associated with subsequent hypoglycemia and mortality in these patients once on dialysis. We studied the associations and further study is needed to establish cause. In addition, more attention is needed in the monitoring of glucose and the use of anti-diabetic medications in DKD patients, especially for those at risk patients.

## Abbreviations

aDCSI: adapted Diabetes Complications Severity Index
CAD: coronary artery disease
CHF: congestive heart failure
COPD: chronic obstructive lung disease
CV: cardiovascular
CVA: cerebrovascular accident
DKD: diabetic kidney disease
DM: diabetes mellitus
ESRD: end-stage renal disease
MI: myocardial infarction
NHIRD: National Health Insurance Research Database
PVD: peripheral vascular disease
T2DM: Type 2 diabetes mellitus
